# Comparison of bone repair of advanced platelet-rich fibrin (A-PRF) and leukocyte- and platelet-rich fibrin (L-PRF) following the extraction of impacted third molars: a randomized clinical trial

**DOI:** 10.1007/s10006-026-01641-6

**Published:** 2026-10-29

**Authors:** Ewerton Rodrigues, Andrea Pontual, Carlos Lago, Ariana Loureiro, Priscilla Pinto, Belmiro Vasconcelos

**Affiliations:** 1 Department of Anesthesiology and Oral and Maxillofacial Surgery, Santo Agostinho University Center, Teresina, Piauí Brazil; 2 Clinical and Preventive Dentistry Department, Oral Radiology Sector, Federal University of Pernambuco, Recife, Pernambuco Brazil; 3 Department of Oral and Maxillofacial Surgery, Coordinator of Postgraduate Program in Oral and Maxillofacial Surgery, Oswaldo Cruz University Hospital, University of Pernambuco, Recife, Pernambuco Brazil; 4 OMS resident, Department of Oral and Maxillofacial Surgery, Oswaldo Cruz University Hospital, University of Pernambuco, Recife, Pernambuco Brazil; 5 Chair of Department of Oral and Maxillofacial Surgery, Coordinator of Postgraduate Program in Oral and Maxillofacial Surgery, Oswaldo Cruz University Hospital, University of Pernambuco, Recife, Pernambuco Brazil; 6 Department of Oral and Maxillofacial Surgery, Federal University of Pernambuco, Oswaldo Cruz University Hospital, R. Arnóbio Marquês, 310 - Santo Amaro, Recife, Pernambuco 50100-130 Brazil

**Keywords:** Bone regeneration, Platelet-rich fibrin, Oral surgery, Postoperative pain, Swelling

## Abstract

**Purpose:**

To compare the effects of leukocyte- and platelet-rich fibrin (L-PRF) and advanced platelet-rich fibrin (A-PRF) on bone healing and postoperative outcomes following impacted third molar extraction (ITME).

**Methods:**

A randomized, double-blind, split-mouth clinical trial was conducted in patients undergoing bilateral ITME. One extraction socket received L-PRF and the contralateral socket received A-PRF. The primary outcome was bone volume at the extraction socket, assessed by cone-beam computed tomography (CBCT), as a measure of bone healing. Secondary outcomes included trabecular thickness, trabecular spacing, soft tissue healing assessed using the modified Landry healing index, postoperative pain assessed using a visual analog scale (VAS), and facial swelling. Statistical significance was set at *p* < 0.05.

**Results:**

Thirty-five patients were included in the final analysis. No significant differences were observed in bone volume between the L-PRF (21.04 ± 7.46) and A-PRF (21.43 ± 7.65) groups (*p* = 0.802). Trabecular thickness was significantly greater in the A-PRF group (*p* = 0.012), whereas trabecular spacing did not differ between groups. An exploratory time-specific comparison showed lower pain scores in the L-PRF group at 8 h postoperatively (*p* = 0.046), although no significant overall treatment effect was observed for postoperative pain. No significant differences were identified regarding facial swelling or soft tissue healing.

**Conclusion:**

Within the limitations of this randomized clinical trial, L-PRF and A-PRF demonstrated comparable effects on bone healing and postoperative clinical outcomes following impacted third molar extraction.

Trial registration Brazilian registry of clinical trials [ReBEC] number: RBR-10vc6r63.

## Introduction

Impacted third molar extraction (ITME) is one of the most commonly performed procedures in oral and maxillofacial surgery [1]. Pain, swelling, trismus, and periodontal defects distal to the second molars are common after surgery, whereas complications such as alveolar osteitis, nerve injury, and bleeding occur less frequently [2]. These conditions may impair daily activities and negatively affect patients’ oral health–related quality of life.

Several approaches have been proposed to reduce postoperative morbidity and improve bone and soft tissue healing, including corticosteroids, photobiomodulation therapy, bone grafts, and autologous platelet concentrates [3–6].

Autologous platelet concentrates have been used to support tissue repair and bone regeneration through the release of bioactive molecules [7, 8]. Platelet-rich fibrin (PRF), a second-generation platelet concentrate obtained without anticoagulants, promotes wound healing, tissue regeneration, and modulation of the inflammatory response [7, 9, 10].

Different PRF derivatives and centrifugation protocols have been developed over the years, including advanced platelet-rich fibrin (A-PRF) [11]. A-PRF is prepared using lower centrifugation speeds and longer centrifugation times than L-PRF, which results in a distinct fibrin architecture — characterized by a softer, less dense matrix — and allows a greater number of leukocytes, including monocytes and neutrophils, to be distributed throughout the clot rather than concentrated at the buffy coat layer [11]. This modified centrifugation protocol has been associated with significantly higher release of key growth factors, including platelet-derived growth factor (PDGF), transforming growth factor-β1 (TGF-β1), and vascular endothelial growth factor (VEGF), compared with both PRP and conventional PRF [3, 11]. Despite these biological advantages, clinical findings regarding the effects of A-PRF on postoperative outcomes after impacted third molar extraction remain inconsistent across studies [4–6].

Although systematic reviews have assessed the use of PRF derivatives after mandibular third molar extraction [5, 6], direct comparisons between L-PRF and A-PRF remain scarce in the literature. Most available studies have evaluated A-PRF against control groups without PRF [4, 8] or focused on a limited set of outcomes, with few studies simultaneously assessing bone healing, soft tissue repair, postoperative pain, and swelling [5]. Additionally, the impact of fibrin architecture differences between L-PRF and A-PRF on clinical outcomes following impacted third molar extraction has not been adequately investigated. The null hypothesis was that no significant differences would exist between L-PRF and A-PRF regarding bone healing, soft tissue healing, postoperative pain, and facial swelling following impacted mandibular third molar extraction. The alternative hypothesis was that A-PRF would provide superior clinical outcomes. Therefore, this randomized clinical trial aimed to compare the effects of L-PRF and A-PRF on bone healing, soft tissue healing, postoperative pain, and facial swelling after impacted mandibular third molar extraction.

## Materials and methods

### Study design

**Fig. 1:**
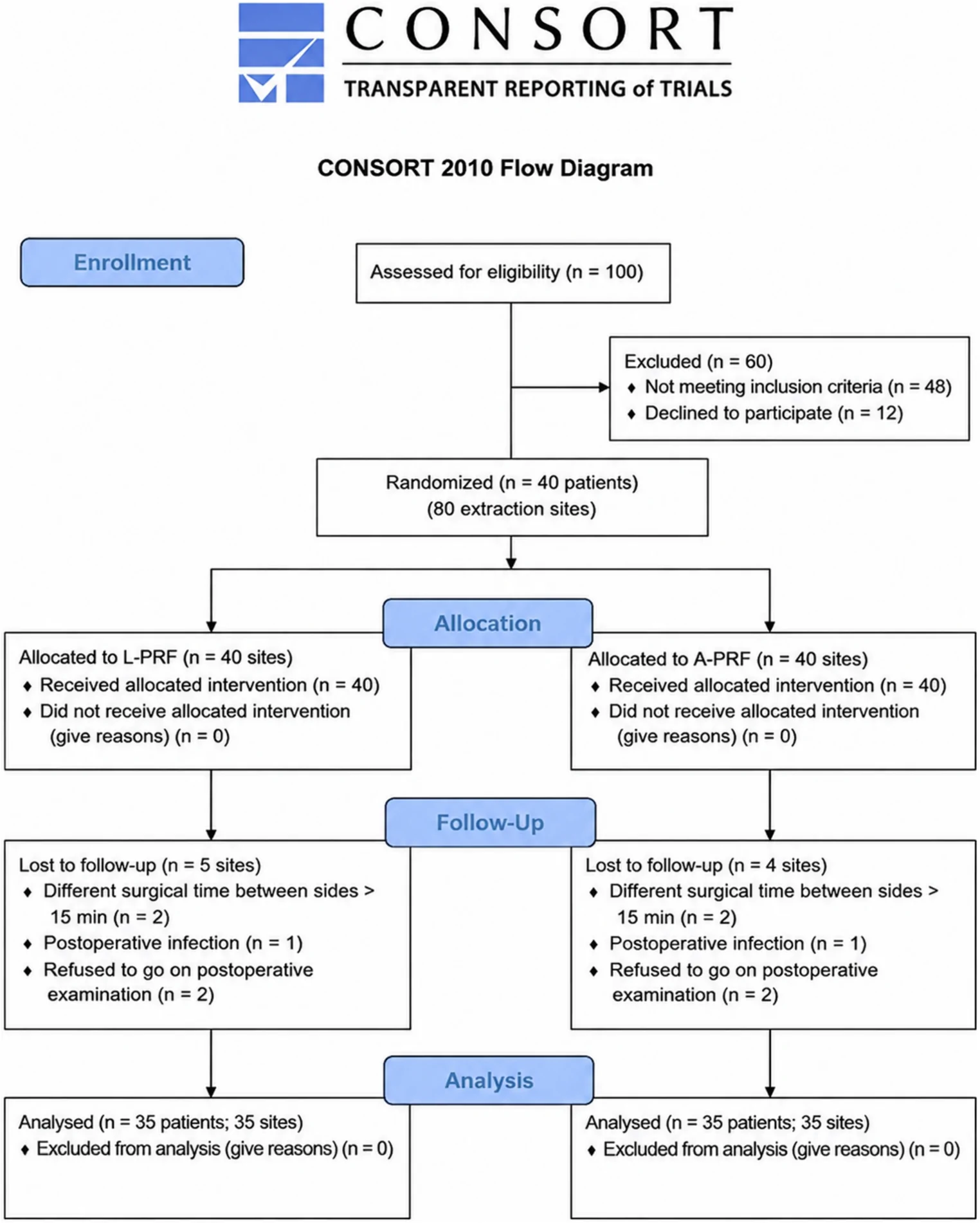
CONSORT flow diagram of participant recruitment, allocation, follow-up, and analysis

This randomized, split-mouth, double-blind clinical trial compared advanced platelet-rich fibrin (A-PRF), considered the experimental intervention, with leukocyte- and platelet-rich fibrin (L-PRF), used as the active control intervention. The study was approved by the Local Research Ethics Committee of the University of Pernambuco, Brazil (approval number: 4,863,219) and registered in the Brazilian Registry of Clinical Trials (ReBEC; RBR-10vc6r63). The study was conducted and reported in accordance with the Declaration of Helsinki and CONSORT guidelines (Fig. 1) [12]. In this study, A-PRF was considered the experimental intervention, given its more recently developed centrifugation protocol and proposed enhanced biological properties, whereas L-PRF was used as the active comparator. The study was conducted under a double-blind design. Participants, the clinical examiner responsible for postoperative assessments (P.S.P.), the oral and maxillofacial radiologist responsible for CBCT analyses (A.A.P.), and the statistician (B.C.E.V.) were blinded to treatment allocation throughout the study.

### Participants and inclusion criteria

The study was conducted at the Clinical Research Center of the Department of Oral and Maxillofacial Surgery and Traumatology, University of Pernambuco, Recife, Brazil. The study was conducted between January 2023 and December 2023. Patient recruitment, surgical procedures, and follow-up assessments were completed within this period. All participants provided written informed consent before enrollment.

Eligible participants were adults aged 18–35 years requiring bilateral mandibular impacted third molar extraction with symmetric impaction patterns and fully developed roots. Patients with systemic diseases, active infection, smoking habits, pregnancy or breastfeeding, use of medications that could interfere with healing, history of allergy to study medications, use of bone-modifying agents, absence of the adjacent mandibular second molar, a time interval greater than 15 min between the two surgical procedures, or failure to complete follow-up were excluded.

### Sample size determination

The sample size calculation was based on the study by Heo et al. [13]. A two-tailed Student’s t-test for independent samples was used to detect a fixed mean difference of 0.10 points, considered clinically relevant, with an assumed standard deviation of 0.05. Based on these parameters, a minimum sample size of 30 patients per group was calculated to achieve a significance level (α) of 0.05 and a statistical power (1 − β) of 0.80. To account for potential dropouts, a total of 40 patients were ultimately enrolled in the study.

### Randomization

Allocation of L-PRF and A-PRF to the surgical sites was performed using a computer-generated randomization sequence created by a single investigator (A.M.L.C.L.) using Microsoft Excel^®^ (Microsoft Corporation, Redmond, WA, USA). The allocation sequence was concealed from patients, the clinical examiner (P.S.P.), the radiologist (A.A.P.), and the statistician (B.C.E.V.) until completion of the statistical analysis.

### Surgical procedure

For the preparation of leukocyte- and platelet-rich fibrin (L-PRF), peripheral venous blood was collected into four 10-mL sterile tubes without anticoagulant and immediately centrifuged at 2700 rpm (approximately 708 × g) for 12 min using a fixed-angle centrifuge (Intra-Spin^®^, BioHorizons, Birmingham, AL, USA). For advanced platelet-rich fibrin (A-PRF), peripheral blood was collected into four sterile plain glass vacuum tubes and centrifuged at 1500 rpm (approximately 208 × g) for 14 min using a dedicated PRF centrifuge (PRF Duo Centrifuge^®^, Process for PRF, Nice, France). Both centrifugation protocols were performed using the centrifuges specifically recommended for each PRF preparation protocol, according to previously validated methods described in the literature [6, 8, 9, 11]. After centrifugation, the PRF clots were separated from the red blood cell fraction and compressed using a standardized PRF box (PRF-Box^®^, Process for PRF, Nice, France) to obtain membranes. Blood collection and centrifugation were performed immediately before surgery to prevent premature coagulation before centrifugation. After membrane preparation, the PRF membranes were maintained under sterile conditions in the PRF Box until placement into the extraction sockets. Given the standardized surgical protocol and the relatively short operative times, this interval was considered unlikely to compromise the biological properties of the PRF membranes.

All surgical procedures were performed by the same experienced oral and maxillofacial surgeon (E.D.R.R.). The same surgical protocol was applied to both study groups, and the only difference between interventions was the type of platelet-rich fibrin membrane placed into the extraction socket (L-PRF or A-PRF). Bilateral mandibular impacted third molars were removed during the same surgical session using a standardized surgical protocol. Preoperative intraoral antisepsis was performed with 0.12% chlorhexidine mouthwash, followed by extraoral antisepsis with 2% chlorhexidine solution. Local anesthesia was achieved using inferior alveolar nerve block associated with buccal and lingual infiltrations with 2% lidocaine containing 1:100,000 epinephrine (Alphacaine^®^, DFL, Rio de Janeiro, Brazil).

A triangular mucoperiosteal flap was elevated, followed by osteotomy and odontosection under copious irrigation with sterile saline solution. After tooth extraction and socket irrigation, four L-PRF membranes or four A-PRF membranes were placed into the extraction socket according to the randomized allocation until complete socket filling was achieved. Wound closure was performed using 4 − 0 nylon sutures (Technofio^®^, Goiânia, Brazil).

All participants received standardized postoperative instructions. A 0.12% chlorhexidine mouthwash was prescribed twice daily for 7 days, and paracetamol (acetaminophen) 750 mg was prescribed as needed for postoperative pain control. No postoperative antibiotics were routinely prescribed.

### Assessment and follow-up

**Fig. 2:**
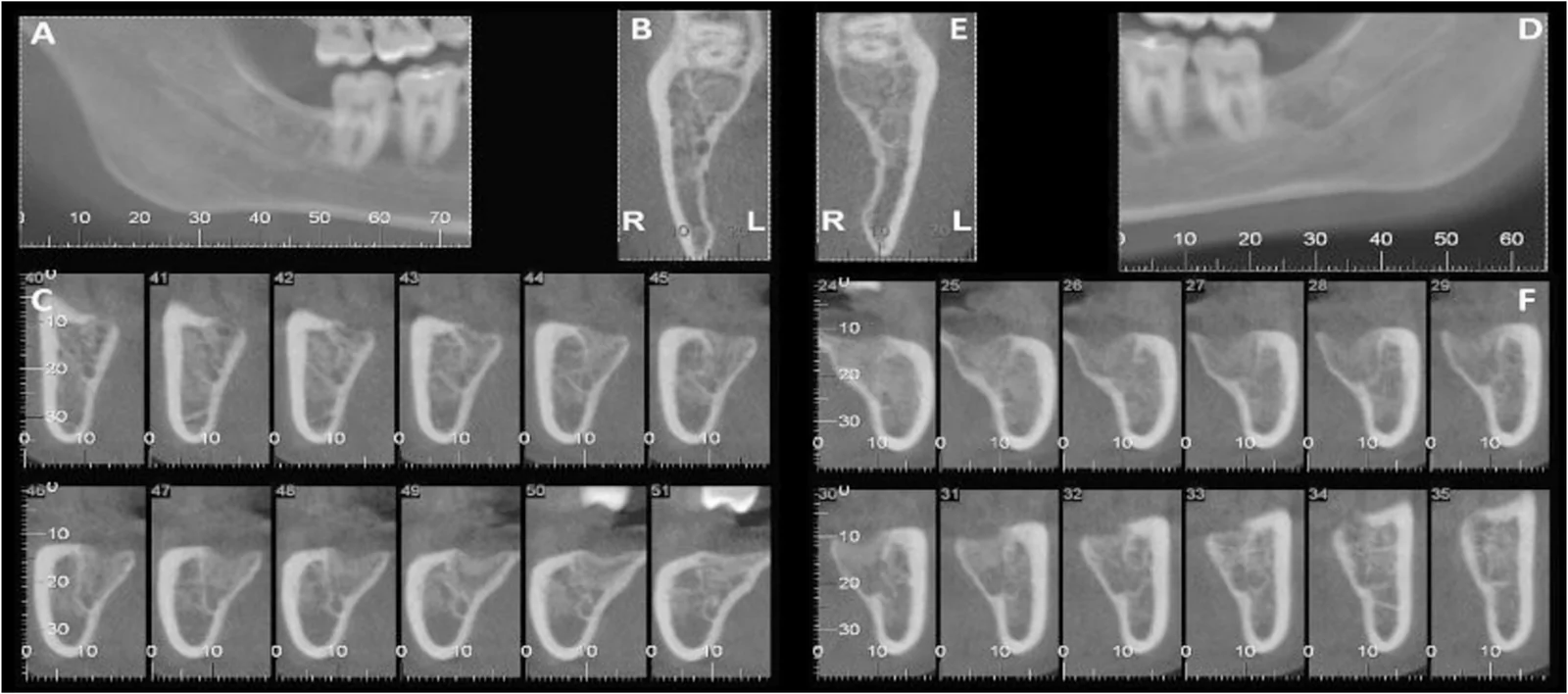
CBCT assessment of bone healing three months after surgery (T3) using the BoneJ plugin to evaluate bone volume, trabecular thickness, and trabecular spacing. Panoramic (**A**), axial (**B**), and parasagittal (**C**) reconstructions of an L-PRF socket and axial (**D**), panoramic (**E**), and parasagittal (**F**) reconstructions of an A-PRF socket from the same patient

The primary outcome was bone volume at the extraction socket, assessed by cone-beam computed tomography (CBCT) three months postoperatively. Secondary outcomes included trabecular thickness, trabecular spacing, soft tissue healing, postoperative pain, facial swelling, and operative time. Demographic data, including age, sex, and impaction pattern according to the Pell and Gregory [14] and Winter [15] classifications, were also recorded. CBCT imaging was performed using the i-CAT Next Generation system (Imaging Sciences International, Hatfield, PA, USA), operating at 120 kVp and 18.5 mAs, with a field of view (FOV) of 6 × 16 cm and a voxel size of 0.25 mm. CBCT scans were obtained three months after surgery (T3) and exported for analysis using ImageJ/Fiji software (National Institutes of Health, Bethesda, MD, USA). All CBCT assessments were performed by an oral and maxillofacial radiologist with 10 years of professional experience. Image registration was performed using the Registration/Rigid 3D tool. A cubic volume of interest (VOI), measuring 4 mm in height, 4 mm in width, and 4 mm in depth, was defined within the medullary bone at the central and basal regions of the extraction socket. Standardization of the VOI across all samples was achieved using the ROI Manager function in ImageJ/Fiji. All images were converted to 8-bit format and binarized using the automatic “Moments” thresholding method [16, 17]. Quantitative analysis of bone volume, trabecular thickness, and trabecular spacing was performed using the BoneJ plugin. In addition, histogram analysis was used to calculate the mean and standard deviation of grayscale values within each VOI (Fig. 2).

Postoperative pain was assessed using a visual analog scale (VAS) consisting of a 100-mm horizontal line, where 0 represented no pain and 100 represented the worst imaginable pain. Patients were instructed to record their pain intensity at 30 min and at 2, 4, 6, 8, 12, 16, 24, 48, and 72 h, and 7 days postoperatively using a standardized assessment form.

**Fig. 3:**
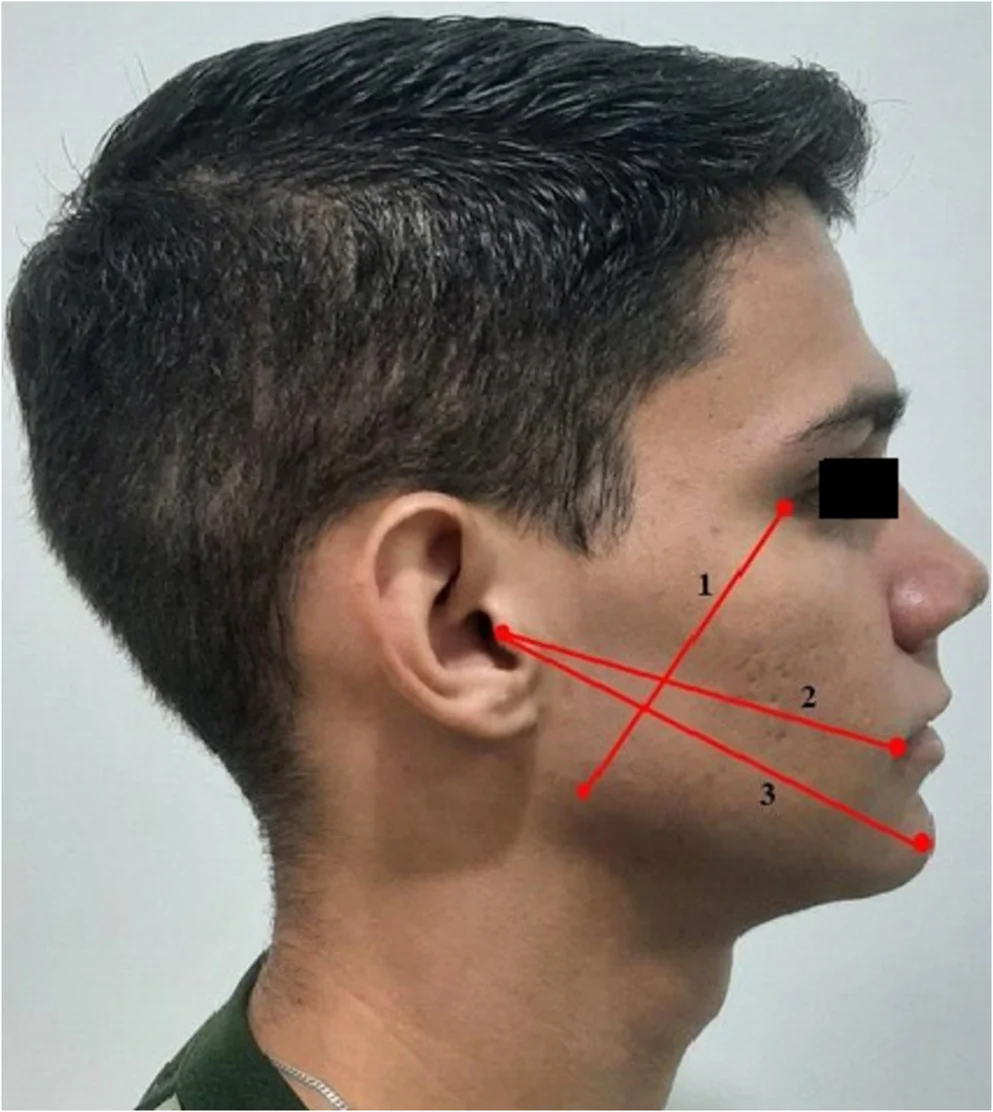
Swelling measurements

Postoperative edema was evaluated by measuring three facial linear distances: (1) from the lateral canthus of the eye to the gonial angle of the mandible; (2) from the inferior border of the tragus to the oral commissure; and (3) from the inferior border of the tragus to the pogonion (Fig. 3).

These measurements were obtained preoperatively (T0) and at 72 h (T1) and 7 days (T2) postoperatively by the same clinical examiner, who was blinded to treatment allocation. Standardized anatomical landmarks and the same measurement protocol were used at all assessment time points to minimize measurement variability. The sum of the three linear measurements was used to quantify the degree of postoperative swelling. At T2, postoperative soft tissue healing was clinically assessed using the modified Landry healing index [8].

### Data analysis

Qualitative variables were described using absolute and relative frequencies, whereas quantitative variables were expressed as means and standard deviations. The Kolmogorov–Smirnov test was used to assess data normality. Because this was a randomized split-mouth clinical trial, statistical analyses accounted for the paired nature of the observations. Comparisons between interventions were performed using the paired Student’s t-test for normally distributed variables and the Wilcoxon signed-rank test for non-normally distributed variables. Postoperative pain scores were analyzed using a two-way repeated-measures analysis of variance (ANOVA), with treatment (L-PRF and A-PRF) and postoperative time as within-subject factors. The treatment × time interaction was included in the model to assess whether changes in postoperative pain over time differed between interventions. The sphericity assumption was assessed for repeated measurements, and Greenhouse–Geisser correction was applied when sphericity could not be assumed. The Greenhouse–Geisser epsilon (ε) was 0.355 for the main effect of time and 0.376 for the treatment × time interaction. Exploratory pairwise comparisons between treatments at individual postoperative time points were performed using the Wilcoxon signed-rank test without formal adjustment for multiplicity. All analyses were performed using SPSS version 20.0 (IBM Corp., Armonk, NY, USA), and statistical significance was established at *p* < 0.05.

## Results

A total of 40 participants met the inclusion criteria. During follow-up, two patients were excluded due to a time interval greater than 15 min between surgical procedures, one developed postoperative infection, and two were lost to follow-up (Fig. 1). Therefore, 35 patients were included in the final analysis.

The mean age of the participants was 21.1 ± 2.7 years (range: 18–32 years), and 25 (71.4%) were female. According to the Pell and Gregory classification, the impacted mandibular third molars were classified as class IIA, IB, or IIB. According to Winter’s classification, 17 (48.6%) were mesioangular and 18 (51.4%) were vertical (Table 1). L-PRF was allocated to the right side in 17 patients and to the left side in 18 patients.

**Table 1:** Demographic and clinical data of the participants of the clinical trial

Group				
Variable		L-PRF	A-PRF	*p*-Value
Sex				
Men		10 (28.6%)		
Women		25 (71.4%)		
**Age (mean ± SD)**		21.1 ± 2.7		
**Surgery time (mean ± SD)**		16.49 ± 3.50	16.6 ± 3.15	0.809^a^
**Pell & Gregory Classification**		**n (%)**	**n (%)**	
IIA		15 (42.85)	15 (42.85)	
IB		9 (25.71)	9 (25.71)	
IIB		11 (31.43)	11 (31.43)	
**Winter Classification**				
Mesioangular		17 (48.58)	17 (48.58)	
Vertical		18 (51.42)	18 (51.42)	

SD, standard deviation. ^a^ Paired Student’s t-test

Tomographic analysis showed no significant differences between L-PRF and A-PRF regarding bone volume or trabecular spacing. However, trabecular thickness was significantly greater in the A-PRF group compared with the L-PRF group (*p* = 0.012) (Table 2).

**Table 2:** Measurements of bone volume (BV), trabecular spacing (Tb.Sp) and trabecular thickness (Tb.Th)

Variable	Group		
	L-PRF	A-PRF	*p*-Value
BV (mean ± SD)	21.04 ± 7.46	21.43 ± 7.65	0.802^b^
Tb.Sp (mean ± SD)	2.53 ± 0.81	2.81 ± 1.14	0.372^b^
Tb.Th (mean ± SD)	1.48 ± 0.50	1.72 ± 0.44	0.012^b^

SD, Standard deviation. ^b^ Wilcoxon signed-rank test

**Fig. 4:**
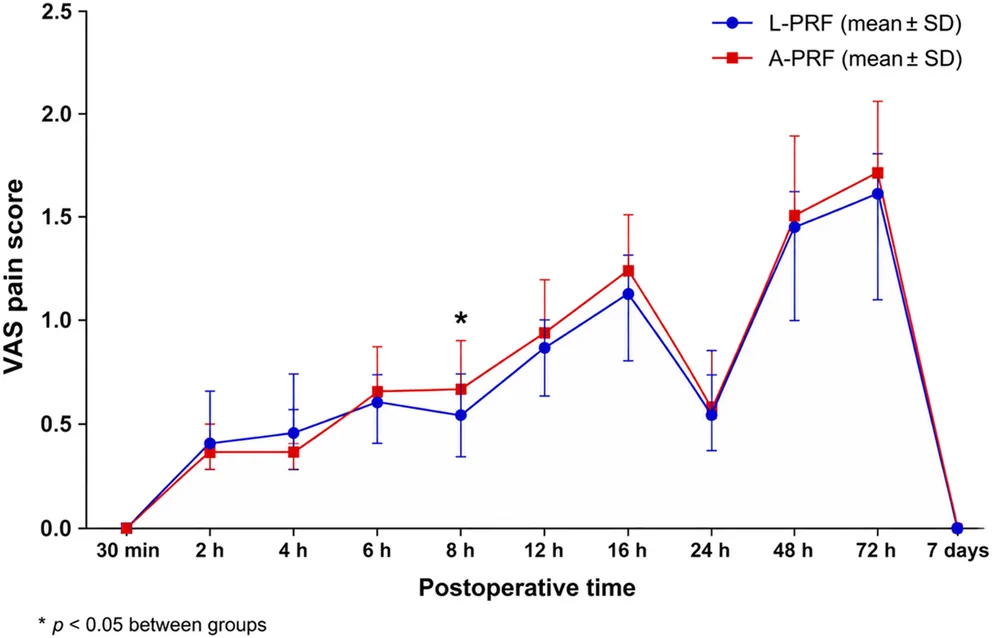
Mean postoperative VAS pain scores following impacted mandibular third molar extraction in the L-PRF and A-PRF groups. Error bars represent standard deviations. *p* < 0.05 for the exploratory pairwise comparison between groups

Postoperative pain scores assessed using the visual analog scale (VAS) are presented in Table 3; Fig. 4. The repeated-measures analysis demonstrated a significant effect of time on postoperative pain (F[3.55, 120.81] = 19.00; Greenhouse–Geisser ε = 0.355; *p* < 0.001), whereas no significant overall treatment effect was observed (F[1, 34] = 0.74; *p* = 0.394). The treatment × time interaction was not statistically significant (F[3.76, 127.90] = 1.84; Greenhouse–Geisser ε = 0.376; *p* = 0.130), indicating that the temporal pattern of postoperative pain did not significantly differ between the L-PRF and A-PRF groups. Exploratory pairwise comparisons at individual postoperative time points showed lower pain scores in the L-PRF group at 8 h (*p* = 0.046), whereas no significant differences were observed at the remaining time points (Table 3).

**Table 3:** Comparison of postoperative pain scores between the L-PRF and A-PRF groups at different time points

Pain level (VAS)			
Postoperative time	L-PRF (mean ± SD)	A-PRF (mean ± SD)	*p*-Value (Wilcoxon signed-rank test)
30 min	0.00 ± 0.00	0.00 ± 0.00	1.000
2 h	0.40 ± 0.50	0.40 ± 0.50	1.000
4 h	0.46 ± 0.61	0.40 ± 0.50	0.317
6 h	0.60 ± 0.69	0.66 ± 0.59	0.414
8 h	0.54 ± 0.78	0.66 ± 0.87	**0.046**
12 h	0.91 ± 0.78	0.94 ± 0.76	0.564
16 h	1.17 ± 0.89	1.26 ± 1.01	0.083
24 h	0.57 ± 0.74	0.57 ± 0.74	1.000
48 h	1.49 ± 1.34	1.57 ± 1.44	0.180
72 h	1.63 ± 1.21	1.74 ± 1.34	0.206
7 days	0.00 ± 0.00	0.00 ± 0.00	1.000

*VAS*, visual analog scale; *SD*, standard deviation; * p < 0.05

Mean postoperative swelling values are presented in Table 4. No significant differences were observed between the L-PRF and A-PRF groups at any postoperative time point.

**Table 4:** Comparison of postoperative swelling and surgical wound healing (Landry index) between the L-PRF and A-PRF groups

Postoperative time	L-PRF (mean ± SD)	A-PRF (mean ± SD)	*p*-Value (Wilcoxon signed-rank test)
Swelling			
Immediate postoperative	96.86 ± 11.81	96.97 ± 11.87	0.353
72 h	101.23 ± 11.70	101.51 ± 12.08	0.160
7 days	96.09 ± 12.13	96.11 ± 12.10	0.990
Landry Index			
7 days	4.29 ± 0.58	4.33 ± 0.64	0.970

SD, standard deviation

At postoperative day 7, modified Landry healing index scores were similar between groups, with mean values of 4.29 for L-PRF and 4.33 for A-PRF.

## Discussion

The present randomized clinical trial compared the effects of L-PRF and A-PRF on bone healing, soft tissue healing, postoperative pain, and facial swelling following impacted mandibular third molar extraction. No significant differences were observed between the two interventions regarding bone volume, trabecular spacing, facial swelling, or soft tissue healing. Although A-PRF demonstrated greater trabecular thickness and L-PRF was associated with lower pain scores at 8 h postoperatively, these isolated findings did not translate into consistent clinical advantages for either intervention. Therefore, the null hypothesis was not rejected, as both platelet concentrates produced comparable outcomes for the evaluated clinical and tomographic parameters.

The inflammatory response following impacted third molar extraction (ITME) can be influenced by multiple factors, including pharmacological management, surgeon experience, extent of surgical trauma, flap design, operative duration, and patient-related variables such as age and sex [8, 18]. In the present study, all surgical procedures were performed by the same surgeon, and no significant differences in operative time were observed between sides. In addition, only third molars with identical impaction patterns and fully developed roots were included, in order to standardize the surgical difficulty and minimize procedural variability.

Previous studies have demonstrated that PRF may accelerate soft tissue healing and reduce postoperative inflammatory responses after impacted third molar extraction [5, 8, 20, 21]. These effects are attributed to the sustained release of growth factors and the presence of a biologically active fibrin matrix that supports tissue repair [9, 18, 22].

Reduced centrifugation forces during A-PRF preparation have been associated with higher concentrations of platelets and leukocytes, as well as increased release of growth factors compared with L-PRF [18, 19]. In the present study, tomographic analysis demonstrated significantly greater trabecular thickness in the A-PRF group, whereas bone volume and trabecular spacing did not differ between groups. Although the increase in trabecular thickness reached statistical significance, its clinical relevance remains uncertain because it was not accompanied by improvements in overall bone volume or in any of the evaluated clinical outcomes, including postoperative pain, facial swelling, and soft tissue healing. Therefore, this isolated radiographic finding should be interpreted with caution until confirmed by further clinical studies.

Both L-PRF and A-PRF have demonstrated the potential to enhance bone healing in experimental and clinical studies [23–26]. In a systematic review of ridge preservation procedures, Balli et al. [27] identified only two randomized controlled clinical trials that met strict eligibility criteria, including CBCT-based assessment. The review reported that L-PRF showed promising effects in reducing horizontal and vertical crestal bone resorption compared with spontaneous healing; however, the authors emphasized the limited evidence base and methodological limitations of the available trials, particularly the small sample size and wide variability reported by Temmerman et al. [25]. These findings highlighted the need for additional well-designed randomized clinical trials evaluating PRF in extraction socket healing. Nevertheless, evidence specifically comparing different PRF protocols remains limited, and direct comparisons between L-PRF and A-PRF after impacted third molar extraction are still scarce [28, 29].

Regarding postoperative pain, lower pain scores were observed in the L-PRF group at the 8-hour postoperative assessment. However, no significant differences were identified between groups at the remaining postoperative time points. In contrast, findings that were also supported by the systematic review by Ramos et al. [30] and the randomized split-mouth study by Caymaz and Uyanik [31].

Postoperative facial swelling was assessed using the sum of three linear facial measurements, a simple and noninvasive method commonly used in oral surgery studies. No significant differences in postoperative edema were observed between groups, which is consistent with previous randomized clinical trials and systematic reviews comparing L-PRF and A-PRF [6, 31]. Although one systematic review reported greater edema reduction with A-PRF on postoperative day 3, no consistent differences were identified across the evaluated time points [6].

Although experimental studies have suggested that A-PRF may enhance angiogenesis and soft tissue healing through increased vascularization and growth factor release [21, 22], these biological advantages were not reflected in the present clinical trial. Consistent with previous clinical evidence [20], both platelet concentrates produced comparable soft tissue healing according to the modified Landry healing index.

Some methodological aspects of the present study deserve consideration. L-PRF was selected as the active control intervention because it is the most extensively investigated platelet-rich fibrin derivative in oral surgery and has demonstrated beneficial effects on healing following impacted third molar extraction. In contrast, A-PRF was considered the experimental intervention due to its modified low-speed centrifugation protocol and proposed enhanced biological properties. In addition, bone volume assessed by CBCT was chosen as the primary outcome because the main objective of the study was to compare the regenerative potential of the two platelet concentrates. Clinical outcomes such as pain, swelling, and soft tissue healing were therefore considered secondary outcomes, as they may be influenced by several patient- and procedure-related factors beyond the biological effects of the platelet concentrates themselves.

Potential sources of bias should also be considered when interpreting the present findings. Selection bias was minimized through the randomized split-mouth design, in which each participant served as their own control. Detection bias was reduced by blinding the clinical examiner, radiologist, and statistician to treatment allocation. Nevertheless, performance bias could not be completely eliminated because the surgeon was aware of the intervention being applied. In addition, the subjective nature of patient-reported pain outcomes may have introduced measurement variability despite the use of a standardized assessment protocol.

This clinical trial has limitations that should be acknowledged. First, the chosen study design involved the extraction of both third molars during the same surgical session, which precluded the independent assessment of postoperative trismus. In addition, pain perception between the two sides may have been influenced by this approach, and the potential impact of rescue analgesic consumption on postoperative pain outcomes cannot be excluded.

Furthermore, pairwise comparisons of postoperative pain were performed at multiple time points without formal adjustment for multiplicity and should therefore be regarded as exploratory. Accordingly, the isolated statistically significant difference observed at 8 h postoperatively should be interpreted with caution, as it may represent a type I error rather than a true treatment effect. This interpretation is further supported by the absence of a significant overall treatment effect or treatment × time interaction in the repeated-measures analysis.

An additional methodological limitation is that the sample size calculation was based on an independent-samples approach and therefore did not explicitly account for the within-subject correlation inherent to the split-mouth design. Although this may have reduced the precision of the sample size estimation, reanalysis using statistical methods appropriate for paired data produced results consistent with the original analyses, supporting the robustness of the study conclusions. In addition, although all clinical and CBCT assessments were performed by the same blinded examiners using standardized protocols, intra-examiner reproducibility was not formally assessed and no reliability coefficient was calculated. Therefore, a certain degree of measurement variability cannot be completely excluded.

Despite these limitations, the split-mouth design offers important methodological advantages by allowing each participant to serve as their own control, thereby reducing interindividual variability. Furthermore, patients, outcome assessors, and the statistician remained blinded throughout the study, strengthening the internal validity and reliability of the findings.

## Conclusion

Within the limitations of the present study, A-PRF and L-PRF demonstrated comparable outcomes regarding bone healing following impacted third molar extraction. No significant differences were observed between the two platelet concentrates regarding facial swelling or soft tissue healing. Although an exploratory time-specific comparison showed lower pain scores with L-PRF at 8 h postoperatively, there was no significant overall treatment effect or treatment × time interaction for postoperative pain.

## Data Availability

No datasets were generated or analysed during the current study.
